# Organization and development of bilateral somatosensory feedback projections in mice

**DOI:** 10.1016/j.isci.2025.112725

**Published:** 2025-05-21

**Authors:** Grace Houser, Alba Vieites Prado, Thomas Topilko, Clara Nguyen, Patricia Gaspar, Nicolas Renier

**Affiliations:** 1 Sorbonne Université, Paris Brain Institute - ICM, INSERM, CNRS, AP-HP, Hôpital de la Pitié Salpêtrière, Paris, France

**Keywords:** Biological sciences, Natural sciences, Neuroanatomy, Neuroscience, Systems neuroscience

## Abstract

Sensory information travels from the periphery to the cortex across relays. Each of these stations receives feedback projections which gate and tune the transmission of feedforward information. We used whole brain imaging combined with viral strategies and single axon tracings to reveal the existence of a bilateral feedback projection from the somatosensory cortex to brainstem relays processing whisker information. Initially, after birth in mice, this feedback loop projects equally to both sides of the brainstem. Then, the projection strengthens preferentially toward the contralateral side while maintaining its ipsilateral component. We found that manipulating the laterality of the feedforward pathway from the brainstem to the opposite cortex altered the laterality preference of the descending feedback projections. This suggests a dynamic interplay between ascending and descending pathways in shaping sensory processing. This study highlights the presence of bilateral integration within the somatosensory system’s feedback projections.

## Highlights

• 3D mapping in mice of the barrel cortex projections to the brainstem
• These projections are bilateral with a contralateral bias emerging from P7 onwards
• The contralateral bias is independent of evoked activity
• The contralateral bias is modulated by the laterality of feedforward pathways

## Graphical abstract

**Figure:**
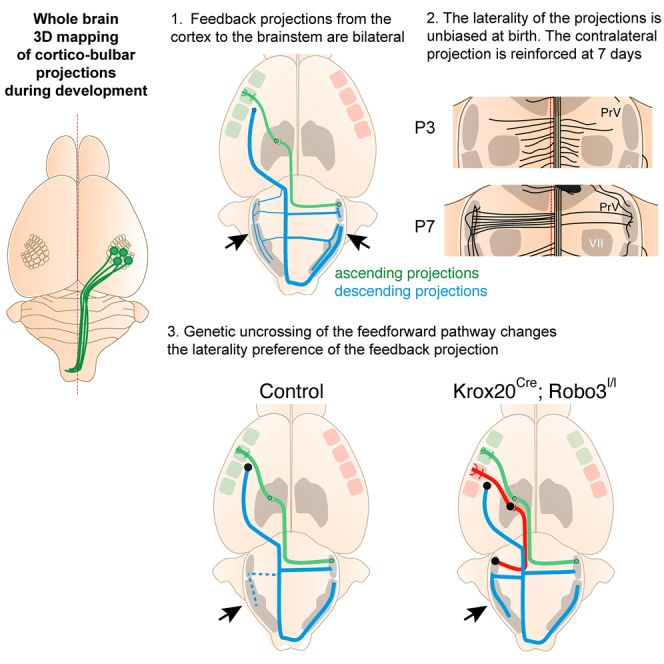

## Introduction

The brain processes sensory information through a complex network of circuits organized across relays. Sensory stimuli travel across multiple stations from the periphery to integrative brain regions via “feedforward” pathways. Another set of connections, called “feedback projections”, carries information back from the integrative centers to the primary sensory relays, contributing to shape the incoming signals. The functional importance of feedback projections has long been recognized as a key feature of sensory processing in the visual,^1^ somatosensory,^2^^,^^3^^,^^4^ olfactory,^5^ and auditory^6^ systems. Feedback projections are important for refining the transmission of sensory inputs based on context, as well as making predictions about upcoming stimuli.

In the somatosensory system, feedforward touch and proprioceptive information from the face is collected by the neurons of the trigeminal complex in the brainstem. This information is then transferred across several stations to the contralateral cortex.^7^ Neurons of the trigeminal complex in the brainstem receive a feedback connection directly from the primary somatosensory cortex, originating from layer 5 pyramidal neurons, which is part of the corticobulbar projection system.^8^^,^^9^^,^^10^^,^^11^ This feedback loop is important to finely tune and gate somatosensory information coming from the whiskers.^12^

Initial studies suggested that this corticobulbar feedback projection is entirely crossed (its axons traveling along the corticospinal tract), meaning that it respects the laterality of the ascending sensory information.^9^ However, in the kitten brain, the presence of a transient ipsilateral projection from the somatosensory cortex to the trigeminal complex was documented during the first postnatal week, which is then lost in the following weeks.^13^ Moreover, the recent availability of large-scale 3D tracing data reveals that in the mouse, a small ipsilateral component is maintained throughout adulthood.^14^^,^^15^ To clarify this, we aimed to revisit the known organization of the corticobulbar feedback projections in the mouse, and particularly the developmental plasticity of these projections, which could indicate how sensory information participates to the establishment and maintenance of these connections.

Recent advances in whole brain optical clearing and light sheet imaging are giving an opportunity to revisit the organization of the cortical feedback descending projections with precise axon mapping tools.^15^^,^^16^^,^^17^^,^^18^

To test the developmental dependence of the laterality of inputs with the laterality of the feedback projections, we used a previously reported mouse model in which trigeminothalamic projections are partially uncrossed.^19^ In this model, the ascending tracts are split into contralateral and ipsilateral components, which lead to the formation of segregated ipsi- and contralateral topographic maps of the mouse face in the thalamus and in the cortex.

In the present study, we characterized the anatomy and the development of the descending corticobulbar feedback projections in the brainstem. We also tested the influence of input laterality on their developmental plasticity. We report that the mouse trigeminal complex is innervated by bilateral feedback projections, with ipsilateral projections that are present throughout the life of the animal. During development, the laterality of these projections is at first unbiased, with a contralateral bias appearing from P7 on. We show that the laterality of the feedback projections is influenced early on by the laterality of the trigeminothalamic and the thalamocortical inputs. This suggests the existence of yet unknown activity-dependent axon guidance mechanisms controlling the collateralization of long-range projections during development.

## Results

### The corticobulbar feedback projection connects bilaterally to the trigeminal complex

**Figure 1:**
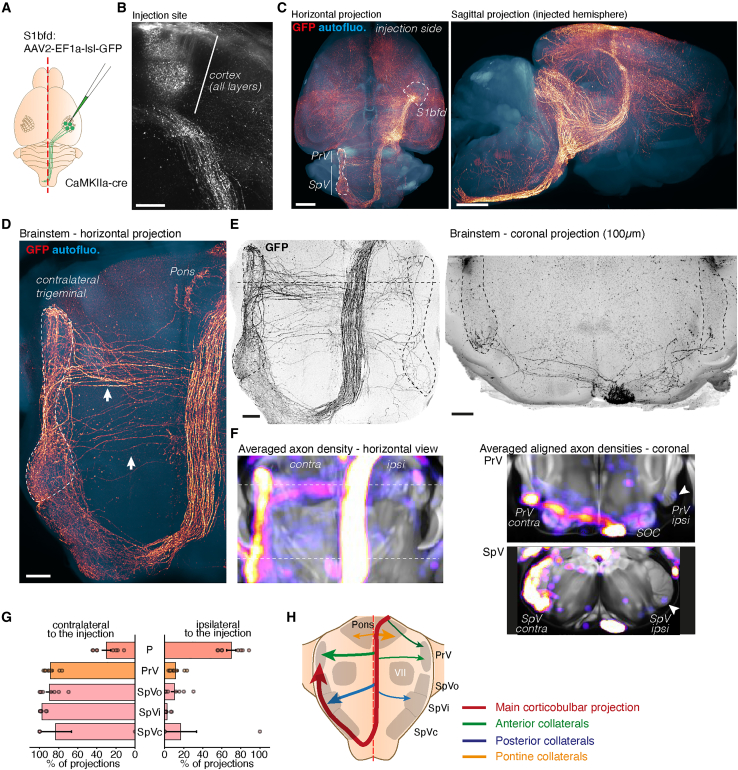
Descending axons from the barrel cortex project bilaterally to the brainstem via collaterals (A) Viral tracing for the visualization of efferent projections from the barrel cortex. iDISCO+ processed adult mouse brain, labeled for GFP (B–F), injected as in (A). (B) Oblique projection at the level of the injection site in the barrel cortex, showing labeled neurons across all cortical layers, and corticofugal axons. (C) Visualization of efferent projections from the barrel cortex: CaMKIIa-cre mice were injected with a cre-dependent AAV and processed with iDISCO+. 3D projections are shown for the GFP+ axons (red) and tissue autofluorescence for context (blue) in sagittal and horizontal orientations, showing the extent of these projections throughout the brain. (D and E) Details of the descending collaterals in the brainstem, shown in horizontal and coronal orientations. (F) TrailMap segmentation of axons, aligned to the CCFv3 reference atlas with ClearMap. Averaged density map of 7 brain shown in sagittal and coronal orientations. (G) Quantification of the laterality of axon densities in the brainstem target nuclei of the corticobulbar projection. Bilateral projections exist for each target. Only the Pontine formation (P) has a majority of ipsilateral projections, while the trigeminal complex receives for the most part contralateral inputs from the cortex. (H) Scheme of the laterality of brainstem collaterals of descending projections. Scale bars are 300 μm (B, D, and E) or 1 mm (C). Data are presented as mean ± SEM.

To visualize corticofugal feedback projections to the brainstem originating from the barrel cortex, we injected a viral tracer expressing a cre-dependent GFP in CaMKII1a-cre adult mice and processed the brain with iDISCO+ with GFP immunolabeling to perform light sheet imaging of the entire projections (Figure 1A, *n* = 7). We validated the injection sites by using the autofluorescence signal in the cortex to delineate the different fields of the somatosensory cortex (Figure S1A). These bulk injections targeted all cortical layers broadly (Figure 1B and S1B). Efferent projections from the barrel cortex were visible as expected in the striatum, motor cortex, contralateral barrel cortex, midbrain (Superior colliculus), thalamus (ventroposteromedial nucleus) and brainstem (Figure 1C). In the brainstem, the projection turned rostrally in a caudal to rostral loop at the level of the inferior olive to enter the contralateral trigeminal nucleus (Figure 1D), consistent with past observations.^9^^,^^11^^,^^12^ In addition to this loop, we documented many collateral axons leaving the descending tract and targeting both sides of the brainstem, with a bias for the side contralateral to the injections (Figure 1E). We quantified the average distribution of axons across several nuclei targeted by these collaterals with TrailMap^18^ and found that the corticobulbar axons consistently entered the trigeminal complexes on both sides, with a strong preference for the contralateral side: 87% ± 3% of contralateral axon pixels in the PrV, and up to 97% ± 3% for the SpVi. Only the Pontine nucleus was strongly innervated by ipsilateral projections (30% ± 5%) (Figure 1F and 1G, *n* = 7). Overall, our data highlight the presence of bilateral collateral fibers projecting from the main corticobulbar tract to the trigeminal complexes on both sides (Figure 1H). These bulk viral tracings show that the descending projections to the trigeminal complex can take multiple paths: contralateral-projecting axons travel mainly via the corticobulbar tract in the brainstem, mixed with the corticospinal tract, and form a caudal loop at the level of the pyramidal decussation to progress in the opposite direction, innervating the contralateral trigeminal complex. Besides this main tract, a set of rostral and caudal collaterals provide additional bilateral projections to the PrV and SpV respectively. The ipsilateral trigeminal complex receives descending cortical projections only via these collaterals, and not via the caudal loop. Additionally, injections in the supplemental somatosensory area (S2), showed that descending projections from S2 also maintained in adults a small ipsilateral component in the brainstem (Figures S1C–S1F, *n* = 3).

**Figure 2:**
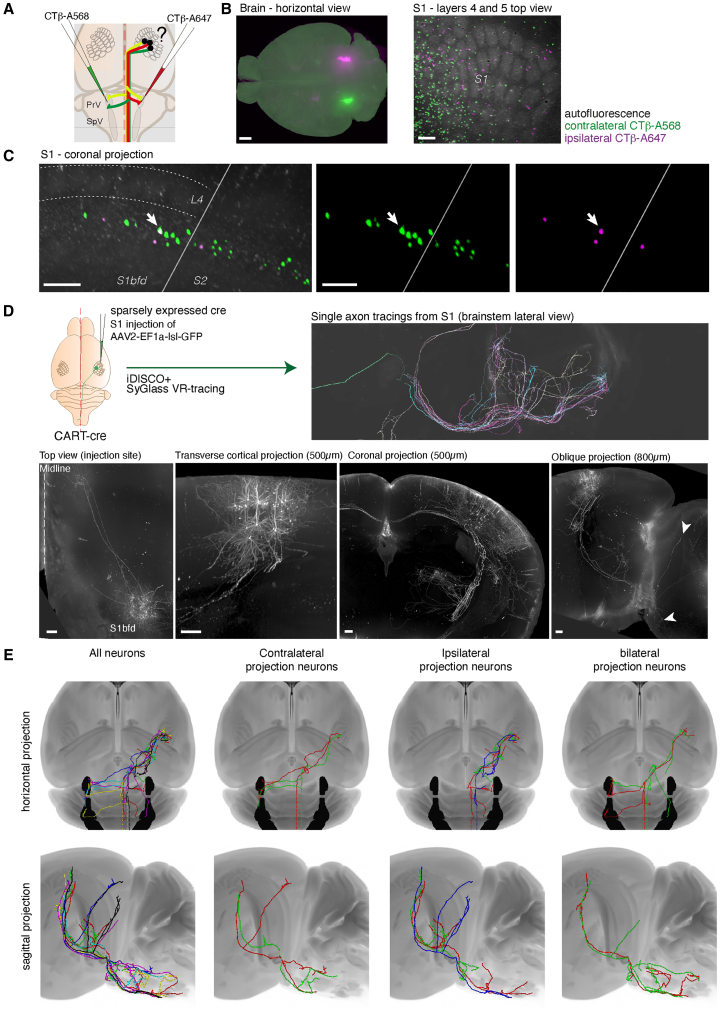
Bilateral collateralization of pyramidal neurons in the brainstem revealed by retrograde tracing and single axon reconstructions (A) Bilateral retrograde tracing of cortical projections to the brainstem with CTβ tracers injected at the level of the PrV nuclei. (B) 3D whole-brain scan of the traced neurons, whole brain horizontal projection (left) and detail of the barrel cortex (right): autofluorescence (gray), contralateral segmented neurons (green) and ipsilateral segmented neurons (magenta). (C) Oblique projection at the level of the barrel cortex, showing the presence of layer 5 CTβ+ cells projecting in majority to the contralateral trigeminal, but also to the ipsilateral trigeminal, and seldomly to both. (D) Use of the CART-cre line to obtain sparse labeling of layer 5 neurons in the barrel cortex. Whole brain scans of AAV-injected brains in the barrel cortex of CART-cre mice show the presence of upper-layer neurons projecting to the motor cortex and corpus callosum, and with a lesser frequency, infra-granular neurons projecting to the CST. Single axons are visible from the cortex down to the brainstem. (E) Virtual-reality assisted reconstructions of seven layer 5 neurons from the barrel cortex projecting to either ipsilateral or contralateral trigeminal nuclei, obtained from the CART-cre line, revealing the different possible trajectories of CST collaterals, as well as the presence of bilaterally projecting neurons. Scale bars are 1 mm (B, left panel) or 200 μm (all other panels).

The presence of ipsilateral projections from the cortex to the trigeminal nucleus was surprising, and not specifically documented previously. Indeed, this projection does not adhere to the expected laterality, based on the ascending tracts. We, therefore, set out to better understand the laterality of projections at the levels of individual neurons. We first performed bilateral retrograde injections at the level of the PrV using cholera toxin β-subunit tracers conjugated with Alexa 568 or Alexa 647, perfused the animals and cleared the brains with iDISCO+ (Figure 2A, *n* = 5 injections over 3 animals). The bilateral injections targeted the rostral parts of the trigeminal complex (Figure 2B). We detected retrogradely labeled neurons bilaterally in the barrel cortex, with 87.5% ± 2.4% of the neurons counted contralateral to the injection, and 12.4% ± 2.4% on the ipsilateral side (*n* = 5 injections) (Figures 2B–2C). On rare occasions, we found neurons labeled with both tracers (1–2 neurons per injection), suggesting the existence of bilaterally projecting neurons.

To confirm the existence of contralateral, ipsilateral, and bilateral projections originating from layer 5 neurons of the barrel cortex, we performed single-axon reconstructions of neurons traced with an anterograde AAV. To obtain sparse labeling of neurons in the primary somatosensory cortex, necessary for single axon tracings, we used the CART-cre transgenic line that we found serendipitously to have a sparse expression pattern in pyramidal neurons of both layers 5 and 3. We generated light sheet scans of the injected brains using iDISCO+, which contained sparse sets of labeled neurons in both supra- and infra-granular layers of the barrel cortex, a few of which projected to the brainstem (Figure 2D). The low density of labeling enabled the reconstruction of the complete axonal trajectories from the cortex to the brainstem, using virtual reality-assisted tracing (SyGlass, IstoVision) (Video S1). We identified neurons with contralateral-projecting collaterals, neurons with ipsilateral collaterals and a few neurons with both (Figure 2E). Most of the traced neuron had several collaterals in the brainstem to the trigeminal complex, to the superior colliculus, or to the anterior pretectal nucleus. Of note, due to technical limitations, the projections were not traced down to the decussation of the pyramids, therefore, their targeting via the caudal SpV loop is missing from these reconstructions (indicated in dotted lines). These reconstructions confirmed the existence of a varied layout of collaterals connecting the cortical pyramidal neurons to the trigeminal complex bilaterally.

Video S1. Tracing of descending projections from CART+ pyramidal neurons of the barrel cortex in adult mice

### The laterality bias of the corticobulbar projection to the trigeminal appears late in development

**Figure 3:**
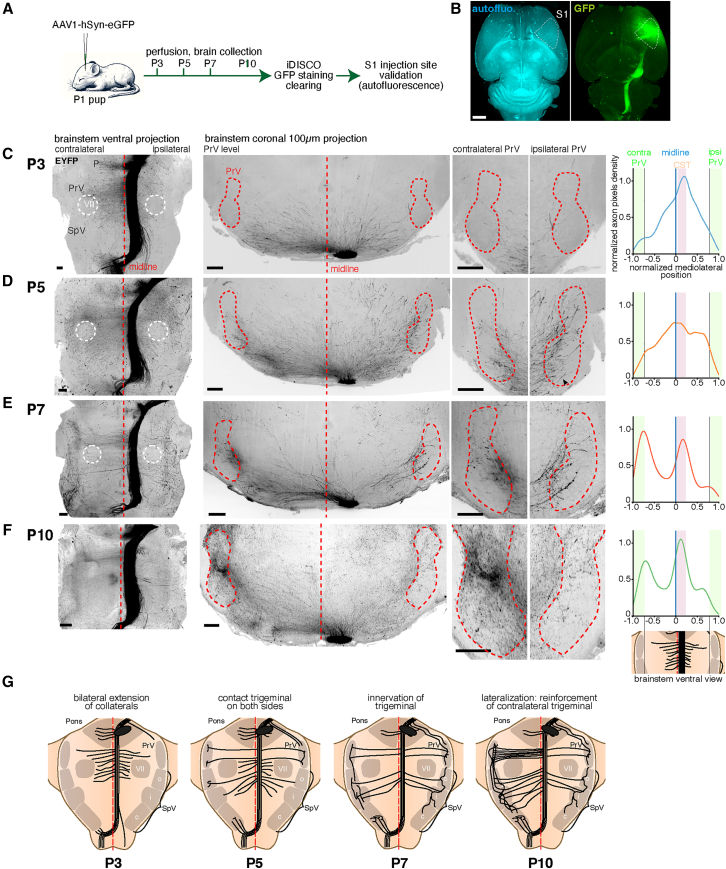
The development of the cortifugal projection to the trigeminal complex is at first unbiased, and then biased toward the contralateral trigeminal complex (A) Experimental timeline for the developmental tracing of corticobulbar projections. (B–F) Whole brain scans of pups injected at P1 with a AAV-EF1a-GFP construct and collected at P3 (C), P5 (D), P7 (E) or P10 (F) (*n* = 3 for each time point, *n* = 4 at P5). (B) Whole brain projection of the labeled tracts (shown here at P7). (C–F) Ventral projections of the brainstem, as well as coronal projections at the level of the PrV. The averaged normalized densities of axon pixels are shown in the right panels as histograms showing the distribution of axons in the mediolateral axis. The mediolateral axis is normalized to the P10 scans to account for tissue expansion. The distributions show a clear peak at the level of the CST, and a peak at the level of the contralateral trigeminal nucleus at P7 and P10. (G) Scheme of the development of corticobulbar collaterals between P3 and P10. Scale bars are 200 μm.

The presence of ipsilateral collaterals could be developmentally regulated and could reflect an incomplete pruning of a more abundant set of early projections, as suggested in kittens.^13^ To test this idea, we analyzed the developmental timing of the corticobulbar projection in the brainstem. We traced descending cortical projections with unilateral AAV injections in the newborn somatosensory cortex. Animals were injected at P1 and sacrificed at P3, P5, P7, or P10 (Figure 3A). The brains were cleared with iDISCO+ and injection sites were checked with the autofluorescence signal (Figure 3B). At P3, the somatosensory corticospinal tract (CST) is already fully extended in the brainstem, with the caudal decussation visible, but not yet reaching the contralateral trigeminal complex (Figure 3C, *n* = 5). Sprouting collaterals were visible on both sides of the midline at all rostro-caudal levels, but the tip of growing axons had not yet entered the trigeminal complex on either side. At P5, invading axons were seen in the trigeminal nuclei on both sides, but the adult pattern of innervation was not yet present. Strikingly, collaterals were present at all rostro-caudal levels, but avoided the location of the facial motor nucleus. At this stage, the density of axons on both sides of the midline was equivalent (Figure 3D, *n* = 4). At P7, both trigeminal nuclei were densely innervated, and a slight imbalance in favor of the contralateral side was noticeable (Figure 3E, *n* = 3). No collaterals were visible at the level of the facial motor nucleus suggesting they have been eliminated between P5 and P7, leaving a ladder-like pattern of collaterals with an anterior group at the level of the PrV and a posterior group at the level of the SpV. At P10, the density of collaterals innervating the trigeminal complex still increased compared to P7, and a strong asymmetry between the contra- and ipsilateral sides was seen (Figure 3F, *n* = 3). This developmental time course shows that the collateralization of the corticobulbar projection coincides with the maturation of the barrel circuits that occur between P3 and P10.^20^^,^^21^^,^^22^^,^^23^ Strikingly, the initial sprouting of corticobulbar axons in newborns is unbiased before reaching their target at P5. Thereafter, between P7 and P10 a contralateral bias occurs and at P10, the organization of the collaterals is reminiscent of the adult pattern, suggesting that most of the ipsilateral-projecting axons are not eliminated by pruning between P7 and adult stages.

### The laterality bias of the descending projections adapts to the laterality of ascending projections, but does not rely on evoked sensory information

The timing of development of the lateralization of collaterals suggests that it may interact with the maturation of network activity in the barrel cortex.^21^ To test this, we performed early deprivations of the whisker sensory inputs by lesioning the infraorbital nerve at P2 and traced the barrel cortex output in adulthood.

**Figure 4:**
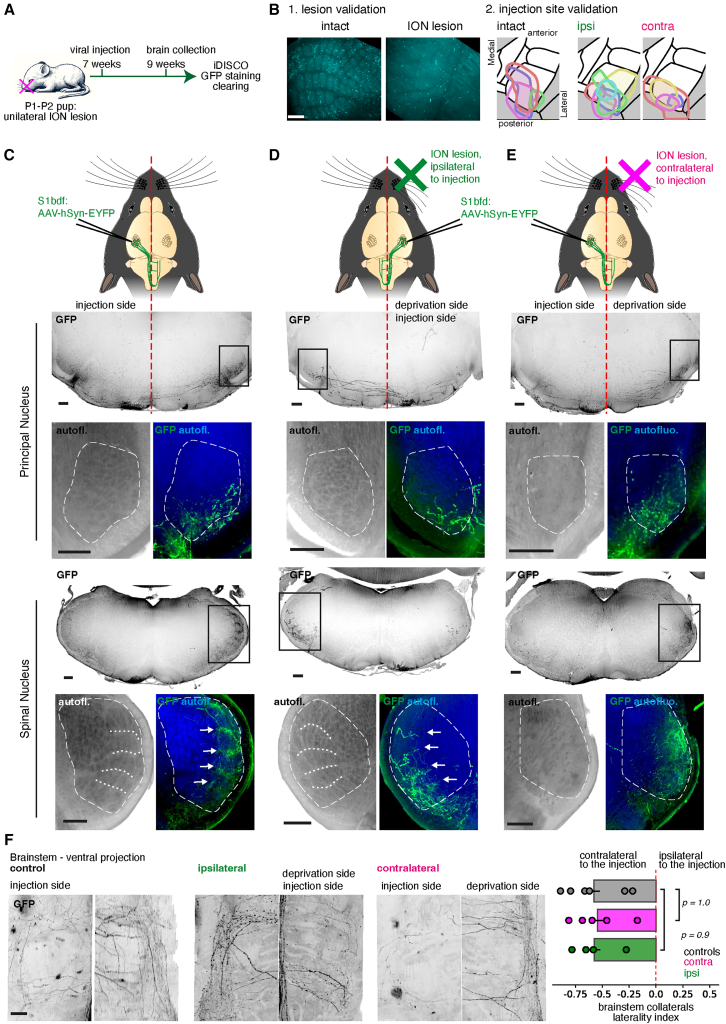
Early sensory deprivation impairs the refinement of descending projections, but not their laterality (A) Experimental timeline of early deprivations and iDISCO+ processing of the GFP labeled brains. (B) Visualization of the effect of the ION lesion in the barrel cortex, showing barrel fusions. Lesioned animals with intact barrels were excluded from the analysis. Spread of labeled neurons at the injection site was verified as well. (C–E) Whole-brain scans of cortical projections to the brainstem in control (C) or P2-deprived (D and E) P30 animals. Coronal projections are shown at the level of the PrV and SpV. Projections to the intact nuclei show a distinctive intra-septal pattern, while projections to the deprived nuclei at both levels are disorganized. (F) Quantification of the laterality-bias of corticofugal axonal densities in control, ipsilateral and contralateral deprived animals. Deprivations have no effect on the laterality ratio of corticofugal projections (*n* = 7 and 5, *p* > 0.9). A Mann-Whitney test was used to calculate the significance of the mean differences. Scale bars are 200 μm.

Unilateral anterograde tracings of barrel cortex neurons were done in adult control and early deprived animals (Figure 4A). Injected brains were then processed with iDISCO+. Using the autofluorescence signal, we checked both the effectiveness of the lesion (leading to barrel fusion) and the spread and accuracy of the injections (Figure 4B). Axons were segmented from the 3D scans with TrailMap. In coronal projections obtained at the levels of the PrV and SpV nuclei, we noted that corticobulbar collaterals preferentially innervated the barrelette septa of both nuclei on the contralateral sides in control animals (Figure 4C, *n* = 6), and in animals ION-lesions ipsilateral to the injection (Figure 4D, *n* = 6). This pattern of septal innervation was not visible in animals with ION lesion contralateral to the injection side, showing that evoked somatosensory inputs are necessary for the correct maturation and fine targeting of corticobulbar axons (Figure 4E, *n* = 6). However, no change in the targeting bias between the deprived and the non-deprived sides was seen between the controls (−0.52 ± 0.06, *n* = 6) and mice with ipsilateral ION lesion (−0.57 ± 0.05, *n* = 4, *p* = 1.0 vs. controls) or contralateral ION lesion (−0.54 ± 0.05, *n* = 6, *p* = 0.9 vs. controls) (Figure 4F). This shows that evoked somatosensory activity, is important for the maturation of the corticobulbar terminal branches in the trigeminal nuclei but is not required for the lateralization of their collaterals.

We, therefore, sought to test whether the lateralization of the descending projections depends on the lateralization of their inputs. We first implemented an injection scheme that would allow us to specifically target cortical neurons of layer 5. The corticobulbar projecting neurons also send projections to other ascending somatosensory relays.^8^ Therefore, we used a dual AAV system consisting of a retrograde virus carrying a bicistronic construct expressing both mCherry and the recombinase FLPo. This injection was combined with an anterograde Flp-dependent virus expressing GFP to label projections targeting the superior colliculus (SC) (Figure S2A). The SC represents the largest target site of layer 5 pyramidal corticofugal axons^8^ receiving contralateral projections from the trigeminal nucleus. The labeled neurons were restricted to the cortical layer 5 (Figure S2B). Whole brain imaging of the anterograde (GFP+) and retrograde (mCherry+) projections revealed a complex bilateral distribution these layer 5 cortical neurons in different nuclei. However, labeled neurons in the trigeminal complex were only seen in the side contralateral to the injection (Figures S2C and S2D). The anterograde tracing from the cortical neurons revealed a pattern of collaterals in the brainstem similar to the one seen in bulk injections (Figures S2E and S2F): 70% ± 4% of contralateral axon pixels in the PrV, and up to 92% ± 3% for the SpVi. This confirmed that dual injections targeting the barrel cortex and superior colliculus can be used to selectively target layer 5 neurons projecting to the trigeminal complex on both sides of the brainstem.

**Figure 5:**
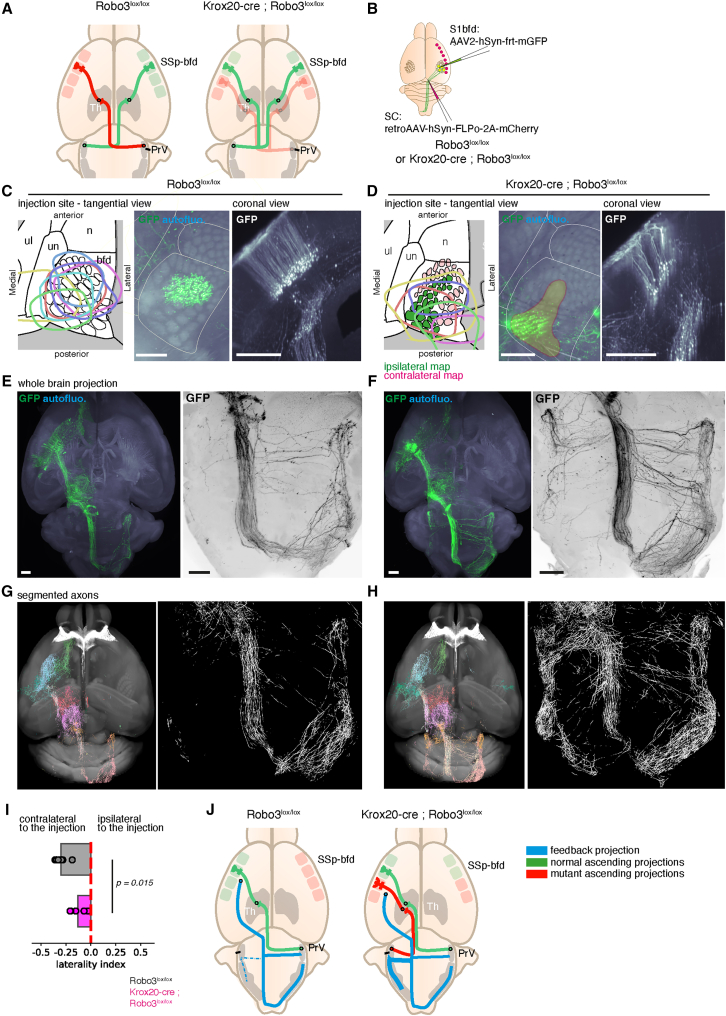
The ipsilateral component of the corticotrigeminal projection is increased in a mouse mutant with an aberrant ipsilateral trigeminal thalamocortical pathway (A) Description of the Krox20-cre; Robo3^lox/lox^ line: afferent projections from the PrV to the thalamic VPM are partially uncrossed. (B) Dual viral AAV injections in the barrel cortex of adult control (C and D) and Krox20-cre; Robo3^lox/lox^ (E and F) mice. (C–F) iDISCO+ processed, GFP labeled, whole brain scans of control (C and E) and Krox20-cre; Robo3^lox/lox^ mutant mice (D and F). Spreads of the injections targeting efferent barrel cortex pyramidal neurons sending collaterals to the ipsilateral Superior Colliculus. In both controls and mutants, labeled neurons were localized in layer 5 (C and D). In Krox20-cre; Robo3^lox/lox^ mutants, an enrichment in ipsilateral collateral projections at the level of the PrV is observed in the horizontal projection at the level of the brainstem (E and F). (G and H) TrailMap segmentation of anterograde axons, aligned with ClearMap (colored by their anatomical location). Detail of the brainstem projections, highlighting the large ipsilateral component in mutant mice. (I) Quantification of the laterality bias in controls and Krox20-cre; Robo3^lox/lox^ mice from TrailMap segmentations, showing a reduced laterality bias in the mutant mice (*n* = 7 and 5, *p* = 0.015). A Mann-Whitney test was used to calculate the significance of the mean differences. (J) Summary diagram of the control and Krox20-cre; Robo3^lox/lox^ mutant organization for the laterality of corticofugal projections. Scale bars are 500 μm.

We then used this assay to test if the laterality of descending corticobulbar projections was dependent on the laterality of the ascending trigeminothalamic and thalamocortical pathways. We turned to a genetic developmental manipulation of the laterality of these ascending tracts. We had previously generated a mutant line, the Krox20-cre; Robo3^lox/lox^ conditional knock out in which afferent trigeminothalamic axons originating from the PrV are partially uncrossed^19^^,^^24^ (Figure 5A). This leads to the formation of a duplicated barrel map in the cortex that respond to inputs from both the contralateral and ipsilateral sides.

We used the dual-AAV injection scheme (Figure S2) to selectively label layer 5 neurons in control and mutant mice. We first verified that in mutant mice, the anterograde projection from the cortex to the superior colliculus was not altered (Figures S3A–S3C). With this dual injection scheme, neurons were labeled as expected in the layer 5 of the barrel cortex in both control and mutant mice. Due to the injection spread, both of the duplicated maps were labeled (Figures 5C and 5D). The proportion of collaterals projecting to the ipsilateral side was higher in mutant mice than control mice. However, the laterality of the branches projecting to the superior colliculus was unchanged (fully ipsilateral as in controls, see Figures S3D and S3E). The increase in corticotrigeminal ipsilateral collaterals in the brainstem was more pronounced in the rostral group at the level of the PrV (Figure 5F). We used ClearMap and TrailMap to quantify the density of labeled axons in the whole brain (Figures 5G and 5H), and quantified the ratio of axonal densities measured in the brainstem. This confirmed a reduced laterality bias in the mutant compared to controls (Controls: 0.30 ± 0.01 toward the contralateral side, mutants: 0.13 ± 0.02 in mutants, *n* = 7 and 5 respectively, *p* = 0.015, Figure 5I). This shows that the laterality of the trigeminothalamic and thalamocortical inputs (which is mixed in the mutant) influences the laterality bias of corticotrigeminal collaterals. These collaterals originated from neurons that also project to the ipsilateral SC, showing that the change of laterality is independently controlled at the level of the targets (Figure 5J).

**Figure 6:**
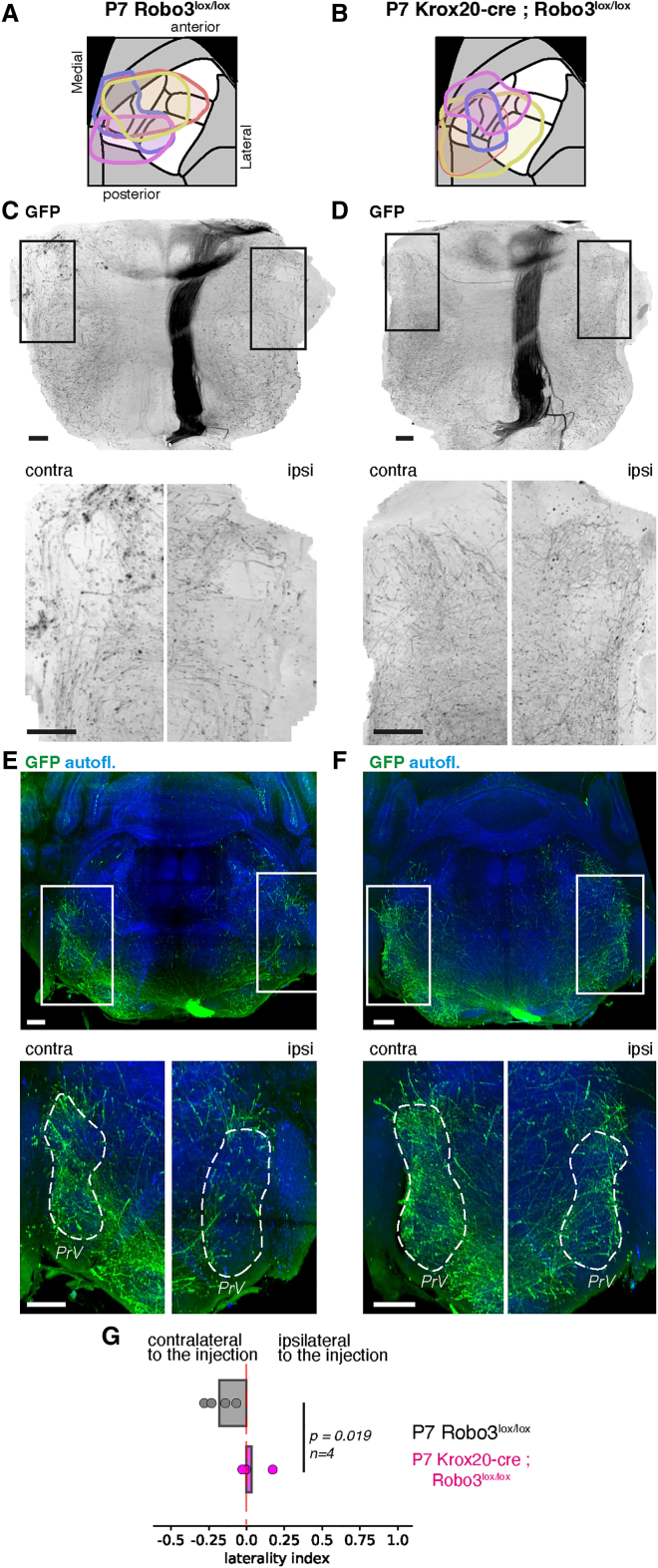
The increase of ipsilateral corticotrigeminal connectivity is visible early on by P7 in the Robo3:KroxCre mouse mutants Whole brain scans obtained from control (A) and Krox20-cre; Robo3^lox/lox^ mice (B) injected with an AAV expressing GFP at P2, and collected at P7. (A and B) Summary of the injection sites and spreads. Owing to the small size of the brain, injections spread over most of S1. (C and D) horizontal ventral projections at the level of the brainstem in control (C) or mutant (D) mice or 100 μm-thick coronal projections at the level of the PrV (C and D). In controls, a small laterality bias is already visible, but axon densities are more evenly distributed across both sides of the midline in mutants. (E and F) Coronal projections (100 μm) at the level of the pontine region, showing the density of innervation of the PrV nucleus in control (E) and mutant (F) mice. In mutant mice, the ipsilateral PrV innervation is denser than in controls. (G) Quantification of the laterality bias in P7 controls and Krox20-cre; Robo3^lox/lox^ mice from TrailMap segmentations, showing a reduced laterality bias in the developing mutant mice (*n* = 4, *p* = 0.019). A Mann-Whitney test was used to calculate the significance of the mean differences. Scale bars are 200 μm.

The altered laterality bias in the mutant could be due to a preferred targeting of the collaterals ipsilaterally during development, or to a selective pruning of contralateral-projecting axons during the maturation of the circuit. To distinguish between these possibilities, we injected a constitutive GFP-expressing AAV tracer in the barrel cortex of control and mutant mice at P1. We harvested the brains at P7 to visualize the descending axons at the beginning of the laterality bias (Figures 6A and 6B). In control mice, the start of an enrichment in contralateral axons was observed at P7, from both the ventral and coronal projections (Figures 6C and 6E). However, in Krox20-cre; Robo3^lox/lox^ mice, the proportion of collaterals was equivalent on both sides of the midline (−0.18 ± 0.02 toward the contralateral side in controls, vs. +0.03 ± 0.02 in mutants, *p* = 0.019, *n* = 4) (Figures 6D, F, and G). This suggests that the initial targeting of corticobulbar collaterals is altered in the Krox20-cre; Robo3^lox/lox^ mice, as early as P7. At this stage, the innervation of the trigeminal complex in wild type mice already shows a small bias toward the contralateral side. Therefore, the higher proportion of ipsilateral axons in mutant mice is more likely the result of an increase in the number of collaterals targeting that side, rather than a decrease in their pruning.

## Discussion

**Figure 7:**
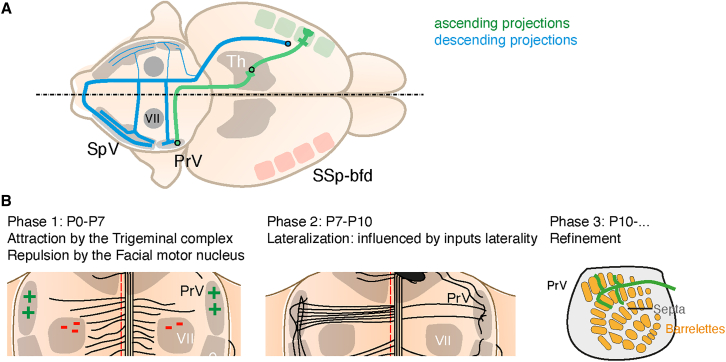
Development and organization of the corticobulbar projection (A) Summary of the developmental findings of this study. (B) Summary of the organization of the main cortico-bulbar projection and its collaterals.

In this study, we found that the descending pathway of the somatosensory cortex mapping the face projects bilaterally to the trigeminal complex of the brainstem with a strong contralateral bias. The layout of this projection in the brainstem is summarized in Figure 7A and can be broken down into 3 phases (Figure 7B): the first phase, during the first postnatal week, where brainstem collaterals grow on both sides of the midline (these axons strongly avoid the facial nucleus). The second phase, during the second postnatal week, when a lateralization bias of the projection appears toward the contralateral side. In this phase, lateralization is reinforced by the addition of collaterals and is influenced by the laterality of ascending projections. The projections are finally refined during a third phase, a process dependent on evoked activity.

Activity-dependent developmental pruning of ipsilateral projections is well-known in the motor corticospinal tract.^25^^,^^26^^,^^27^ However, retained bilateral projections of the motor CST to the spinal cord have been described in adult animals, and are suggested to be important for functional recovery after lesions.^28^ The equivalent findings for the somatosensory CST have not been reported before in mice. Indeed, ipsilateral projections represent a minor proportion of the total projections and as a result only became clearly visible in recent studies using whole-brain 3D reconstructions enabling continuous reconstructions of axonal trajectories.^14^^,^^15^ It is, therefore, possible that, as for the motor CST, the ipsilateral component of the somatosensory CST could support functional plasticity and recovery after lesions.

The role of this projection in sensory processing is still not completely understood. The somatosensory cortex is actively involved in the control of whisking motions.^29^ Processing of whisker positions and motion is in part computed locally by intersubnuclear neurons of the trigeminal complex.^30^^,^^31^ Cortical projections from S1 and S2 to the interpolaris subdivision of the spinal trigeminal nucleus modulate this local computation, before any information is sent to the thalamus.^32^ Tracing studies combined with electrophysiological recordings further confirmed that corticofugal neurons projecting to the trigeminal nuclei are activated by active deflection of the whiskers.^8^ The corticotrigeminal projection also modulates pain perception through a strong excitatory input onto inhibitory neurons of the SpV.^33^ The corticobulbar projections, therefore, participate in context-dependent gating of sensory processing in the brainstem and it will be important to determine if the ipsilateral component of this projection plays a significant role there, therefore, participating in bilateral integration of touch inputs.

The main path taken by descending axons is set by transcription factors^34^^,^^35^^,^^36^^,^^37^ and the expression of guidance cues lining the CST such as Netrin-1, Ephrins, and Semaphorins.^38^^,^^39^^,^^40^^,^^41^^,^^42^ The molecular control of the laterality of CST collaterals has been well established for the motor CST, in part by the studies of genetic models of mice with altered hopping gait. For instance EphrinB3, expressed by the spinal cord midline prevent the midline crossing of the motor CST expressing EphA4.^43^^,^^44^^,^^45^^,^^46^^,^^47^^,^^48^ Our study of a genetic model with partial deletion of Robo3 that creates bilateral somatosensory inputs to the cortex, showed that collaterals in the brainstem adapt their targeting to the side matching ascending inputs very early during development. This suggests the presence of an activity coherence detection mechanism driving the sprouting and orientation of descending collaterals. Pioneer axons would first reach the trigeminal complexes on either side with an unbiased laterality. Our data may suggest the existence of an ipsilateral component to the ascending pathway originating from the brainstem. Our tracing experiments at this point do not support this hypothesis, but more precise investigations may be needed to fully exclude this possibility. Then, axons contacting the trigeminal neurons appear to match their laterality to the ascending trigeminothalamic inputs, which could serve as a scaffold for subsequent collaterals. Thus, the developmental remodeling of this projection could serve as a model to identify an original mechanism linking early neuronal activity and collateralization.

### Limitations of the study

This study is limited to anatomical observations and developmental mechanisms characterized exclusively in mice. As such, these findings might not be directly translatable to other mammalian species, including humans, due to potential differences in developmental timing, sensory processing mechanisms, and connectivity principles, as seen in kittens.^13^ Furthermore, although whole-brain imaging provided comprehensive anatomical mapping, functional correlates of the described anatomical changes were not directly assessed, limiting interpretations regarding physiological roles. Lastly, the genetic model used in this study may introduce confounding developmental compensations that could influence the generalizability of our conclusions.

## Resource availability

### Lead contact

Further information and requests for resources and reagents should be directed to and will be fulfilled by the Lead Contact, Nicolas Renier (nicolas.renier@icm-institute.org).

### Materials availability

This study did not generate new unique reagents.

### Data and code availability

• Raw data are available upon request to the lead contact.
• Codes used for this project are available at DOI: https://zenodo.org/records/3924619. Continuous updates are also posted here: https://github.com/ClearAnatomics.
• Any additional information required to reanalyze the data reported in this paper is available from the lead contact upon request.

## Acknowledgments

We would like to thank all members of the Renier laboratory for insightful discussions and comments. This work was made possible by the Paris Brain Institute core facilities: ICM. Quant imaging core facility, iVector viral core facility, and the ICMice phenopark animal core facility. Our funding sources are: the 10.13039/501100000781European Research Council ERC-Stg NeuroRemod 758817, Paris Emergence(s) award to N.R., 10.13039/501100001665Agence Nationale de la Recherche ANR PRC BaVar, 10.13039/501100001665Agence Nationale de la Recherche Investissement d’avenir ANR-10-IAIHU-06. T.T. is the recipient of a 10.13039/501100002915Fondation pour la Recherche Médicale (FRM) fellowship, and A.V.P. was the recipient of a Marie Sklodowska Curie Action fellow (GA: 845685, LongPlaNet).

## Author contributions

Conceptualization: G.H., A.V.P., and N.R. Methodology: G.H., A.V.P., T.T., P.G., and N.R. Investigation: G.H., T.T., A.V.P., C.N., and N.R. Funding acquisition: N.R. Project administration: N.R. Supervision: P.G. and N.R. Writing – original draft: N.R. Writing – review and editing: all authors.

## Declaration of interests

Authors declare that they have no competing interests.

## STAR★Methods

### Key resources table

**Table:** 

REAGENT or RESOURCE	SOURCE	IDENTIFIER
**Antibodies**		

Rabbit pAb anti-cFos	Synaptic Systems	226003; RRID: AB_2231974
Chicken anti-green fluorescent protein (GFP)	Aves labs	GFP-1020; RRID: AB_10000240
Rabbit anti-red fluorescent protein (RFP)	Rockland	600-401-379; RRID: AB_2209751
Goat anti-hRobo3	R&D systems	AF3076; RRID: AB_2181865

**Bacterial and virus strains**		

pAAV-hSyn-EGFP	Addgene	50465-AAV1; RRID: Addgene_50465
ssAAV-9/2-hSyn1-chI-dFRT-EGFP(rev)-dFRT-WPRE-hGHp(A)	Viral Vector facility - ETH Zurich	V335-9
ssAAV-retro/2-hSyn1-chI-mCherry_2A_FLPo-WPRE-SV40p(A)	Viral Vector facility - ETH Zurich	V173-retro

**Chemicals, peptides, and recombinant proteins**		

Cholera toxin subunit B (recombinant), Alexa Fluor™ 647 conjugate (CTB 647)	Thermo Fisher Invitrogen	C34778
Cholera toxin subunit B (recombinant), Alexa Fluor™ 555 conjugate (CTB 555)	Thermo Fisher Invitrogen	C34776

**Experimental models: Organisms/strains**		

CART-cre mice (B6;129S-Cartpttm1.1(cre)Hze/J)	Jackson Laboratories	Stock 028533; RRID:IMSR_JAX:028533
CamKIIa-cre mice (B6.Cg-TgC amkIIa-cre)T29-1Stl/J)	Jackson Laboratories	Stock 005359: RRID:IMSR_JAX:005359
Krox20:Cre;Robo3lox/lox mice	Mouse Clinical Institute–Institut Clinique de la Souris, Illkirch, France	
RjOrl:SWISS mice	Janvier Laboratories	RRID:IMSR_Rj:SWISS

**Software and algorithms**		

Fiji	Schneider et al., 2012^49^	https://imagej.nih.gov/ij/
Imaris	Oxford Instruments	https://imaris.oxinst.com/
Syglass	IstoVisio	https://www.syglass.io/
ITKsnap	Yushkevich P.A. et al., 2006^50^	http://www.itksnap.org
TubeMap	Kirst et al.^51^	https://github.com/ClearAnatomics/ClearMap
TrailMap	Friedmann et al.^18^	https://github.com/albert597/TRAILMAP
Deeptrace	Gongwer M.W. et al., 2023^52^	https://github.com/DeNardoLab/DeepTraCE
GraphPad	GraphPad software	https://www.graphpad.com/scientificsoftware/prism/

### Experimental model and study participant details

All procedures followed the European legislation for animal experimentation (directive 2010/63/EU). Animal manipulations were approved by the French Ministry of Research and by the local Ethical Committee Darwin-5 (Project 8461-2017010619499922). CART-cre mice, or B6;129S-Cartpttm1.1(cre)Hze/J, and CamKIIa-cre mice (B6.Cg-Tg(Camk2a-cre)T29-1Stl/J) were obtained from Jackson Laboratories (stock 028533 and 005359 respectively), kept as hemizygotes, and back-crossed to C57BL6/NRj in every generation, obtained from Janvier Laboratories. The Robo3 conditional knockout mouse line Krox20:Cre;Robo3lox/lox was established at the MCI/ICS (Mouse Clinical Institute–Institut Clinique de la Souris, Illkirch, France; http://www-mci.u-strasbg.fr) and was generated as previously described.^24^ Controls for experiments using this mouse line were littermate controls lacking the Cre: Robo3lox/lox. The genotypes of these mice were confirmed using PCR. The following primers were used to identify the lox allele sequence for the conditional Robo3: CCAAGGAAAAACTTGAGGTTGCAGCTAG (forward primer) and GATTAGGGGAGGTGAGACATAGGG (reverse primer). The following primers were used to identify the Cre allele sequence for the Krox20Cre: GAT TTC CGT CTC TGG TGT AGC (forward primer) and GCC ATC TTC CAG CAG G (reverse primer).

Mice of the strain RjOrl:SWISS (from Janvier Laboratories) were used for the developmental time course and deprivation experiments.

Upon arrival, the mice were kept in an acclimatation sector for a one-week period to get habituated to our facilities before housing in the general sector. Mice were bred and maintained at the specific pathogen free mouse facility of Paris Brain Institute, with controlled temperature (21 ± 1°C) and humidity (40-70%), *ad libitum* access to standard mouse chow and water, and on a 12h:12h light/dark cycle (lights on at 8AM). Lighting cycles with a controlled rising and decreasing gradient at 8am / 8pm respectively, simulating natural light. Mice were housed in groups of 3-6 during experiments or single housed when nesting abilities were evaluated. Experiments were performed on both male and female animals, randomly assigned. Unless specifically specified in the experiments (eg. For time courses), adult animals included in experimental procedures were aged between 8 weeks and 15 weeks. Mice of both genders were used for all experiments.

### Method details

#### Stereotactic injections

##### Adult mice

Mice were anesthetized with isoflurane (IsoVet): 4% for induction and maintained at 1.8% with an oxygen flow rate of 250 mL/min. Subcutaneous carprofen (Rimadyl®) was administered as 5 mg/kg at a concentration of 50 mg/ml as an analgesia to prevent pain. Then, the fur on the head was shaved, and the skin was cleaned with iodine and alcohol. Ophthalmic ointment was applied to the eyes to keep them from drying out during surgery. The head of the mouse was positioned in a stereotaxic frame (Kopf Instruments, California) and a craniotomy was made above the desired site of injection with a micro drill (0.5 mm tip diameter: FST #19007-05). Targeting of primary somatosensory cortex barrel field was accomplished using the following coordinates: 1.6 mm caudal to bregma, 3.0 mm lateral to the midline, and 0.4 mm below the brain surface. Targeting of the superior colliculus was accomplished using the following coordinates: 4 mm and 3.8 caudal to bregma (RC target), 1 mm and 1.2 mm lateral to the midline (ML target), and 2 mm below the brain surface (DV target). There are 2 different coordinates listed for the rostral-caudal (RC) target and the medial-lateral (ML) target because the superior colliculus was targeted using a grid system of two different coordinates in close proximity. Targeting of the principal sensory trigeminal nucleus (PSV) was accomplished using the following coordinates: 1.0 mm caudal to lambda, 1.9 mm lateral to the midline, and 4.0 mm below the brain surface. The injections were made using the Nanoject III, programmable nanoliter injector (Drummond Scientific Company, USA) and pulled glass capillaries (1.14mm O.D. x 3.5" length x .53mm I.D.: Drummond Scientific Company, USA). Injections made with the AAV viruses were 100 nL volume injections infused at a rate of 1 nL/sec, 10 nL volume cycles, and a 1 sec wait between cycles. Injections made with Cholera toxin subunit B (CTB) were 50 nL volume injections infused at a rate of 1 nL/sec, 10 nL volume cycles, and a 1 sec wait between cycles. After the injection was complete, the capillary was removed 2-5 min later in the case of the AAV injections and 10-15 min later in the case of the CTB injections (the longer wait time was used here because the injection site was deeper). The skin was sutured using Vicryl 6.0R suture material, and the mice were left to recover in a 37°C chamber until the effects of the anesthesia wore off. Analgesic treatment (5 mg/kg of 50 mg/ml carprofen) was administered every 24 hours for a total of 48 hours post surgery.

##### Pups

Mouse pups, P0-P3, were cold-anesthetized. The injections were made through the intact skin and skull using the Nanoject III, programmable nanoliter injector (Drummond Scientific Company, USA) and pulled glass capillaries (1.14mm O.D. x 3.5" length x .53mm I.D.: Drummond Scientific Company, USA). Injections made with the AAV viruses were 100 nL volume injections infused at a rate of 1 nL/sec, 10 nL volume cycles, and a 1 sec wait between cycles. After the injection was complete, the capillary was removed 1-2 min later. The total time the pup spent on ice was less than 10 min. The mouse pups were left to recover in a 37°C chamber until the effects of the anesthesia wore off and they warmed up, to be then return to the mother.

##### Injected toxins and viruses

(1) Cholera toxin subunit B (recombinant), Alexa Fluor™ 647 conjugate (CTB 647) (reference C34778 from Thermo Fisher Invitrogen) was injected unilaterally in the brainstem, at the level of the PSV, of adult mice. Cholera toxin subunit B (recombinant), Alexa Fluor™ 555 conjugate (CTB 55) (reference C34776 from Thermo Fisher Invitrogen) was injected unilaterally and opposite to the CTB 647 in the brainstem, at the level of the PSV, of adult mice. The mice were sacrificed 48 hours after the CTB injection.
(2) The virus pAAV-hSyn-EGFP (Addgene reference: 50465-AAV1) was used as an anterograde tracer in mouse pups and in adult mice (Figures 3, 4, 6, S1, and S3).
(3) The virus ssAAV-retro/2-hSyn1-chI-mCherry_2A_FLPo-WPRE-SV40p(A) (from ETH Zurich) (titer: 7.7 x 10E12 vg/ml) was used as a retrograde tracer and as part of a dual viral system with a conditional anterograde tracer (Figures 5 and S2).
(4) The virus ssAAV-9/2-hSyn1-chI-dFRT-EGFP(rev)-dFRTWPRE-hGHp(A) (from ETH Zurich) (titer: 4.6 x 10E12 vg/ml) was used as a FLP dependent anterograde tracer (Figures 5 and S2).
(5) The virus AAV1-Ef1a-DIO-EYFP (Addgene, reference 27056-AAV1, lot v20736) was used as an anterograde tracer in CaMKIIa-cre and CART-Cre mice (Figures 1 and 2).

#### Intracardiac PFA perfusions

All animals were sacrificed by Pentobarbital overdose (200mg/100g of animal). Intracardiac perfusion was then performed with a peristaltic pump (Gilson, USA). Blood was washed by infusing 30mL of cold PBS followed by tissue fixation with 30mL of cold 4% Paraformaldehyde (Electron Microscopy Sciences, USA) diluted in PBS. Then brain dissection was quickly done taking special care to preserve the brain structure. Finally, the brains were then postfixed for 2 hours at room temperature by immersion in 4% Paraformaldehyde, and stored in PBS at 4°C until further processing.

#### iDISCO+ whole brain immunolabeling

##### General procedure

Whole brain immunostaining was performed following the iDISCO+ protocol, as previously described for vascular studies^16^^,^^51^ with minimal modifications. All the steps of the protocol were done at room temperature with gentle shaking unless otherwise specified. All the buffers were supplemented with 0,01% Sodium Azide (Sigma-Aldrich, Germany) to prevent bacterial and fungal growth.

Perfused brains were dehydrated in an increasing series of methanol (Sigma-Aldrich, France) dilutions in water (washes of 1 hour in methanol 20%, 40%, 60%, 80% and 100%). An additional wash of 2 hours in methanol 100% was done to remove residual water. Once dehydrated, samples were incubated overnight in a solution containing a 66% dichloromethane (Sigma-Aldrich, Germany) in methanol, and then washed twice in methanol 100% (4 hours each wash). Samples were then bleached overnight at 4°C in methanol containing a 5% of hydrogen peroxide (Sigma-Aldrich). Rehydration was done by incubating the samples in methanol 60%, 40% and 20% (1 hour each wash). After methanol pretreatment, samples were washed in PBS twice 15 minutes and 1 hour in PBS containing a 0,2% of Triton X-100 (Sigma-Aldrich) and further permeabilized by a 24 hours incubation at 37°C in Permeabilization Solution, composed by 20% dimethyl sulfoxide (Sigma-Aldrich), 2,3% Glycine (Sigma-Aldrich, USA) in PBS-T. In order to start the immunostaining, samples were first blocked with 0,2% gelatin (Sigma-Aldrich) in PBS-T for 24 hours at 37°C, the same blocking buffer was used to prepare antibody solutions. Primary antibodies were incubated for 10 days at 37°C with gentle shaking, then washed in PBS-T (twice 1 hour and then overnight), and finally newly incubated for 10 days with secondary antibodies. Secondary antibodies raised in donkeys, conjugated to Alexa 647 were used (Life Technologies). After immunostaining, the samples were washed in PBS-T (twice 1 hour and then overnight), dehydrated in a methanol/water increasing concentration series (20%, 40%, 60%, 80%, 100% one hour each and then methanol 100% overnight), followed by a wash in 66% dichloromethane – 33% methanol for 3 hours. Methanol was washed out with two final washes in dichloromethane 100% (15 min each) and finally the samples were cleared and stored in dibenzyl ether (Sigma-Aldrich) until light sheet imaging.

##### Antibodies

Rabbit pAb anti-cFos (Synaptic Systems, 226003) at a dilution of 1:2000.

chicken anti-green fluorescent protein (Aves labs, SKU: GFP-1020) at a dilution of 1:3000. rabbit anti-RFP (Rockland, item no. 600-401-379) at a dilution of 1:3000. Goat anti-hRobo3 (R&Dsystems, AF3076) at a dilution of 1:500. Secondary antibodies made in donkey and conjugated to Alexa dyes were obtained from Jackson Immunoresearch and used at 1μg/mL.

#### Light sheet microscopy

The acquisitions were done on a LaVision Ultramicroscope II equipped with infinity-corrected objectives. The microscope was installed on an active vibration filtration device, itself put on a marble compressed-air table. Imaging was done with the following filters: 595/40 for Alexa Fluor-555, and -680/30 for Alexa Fluor-647. The microscope was equipped with the following laser lines: OBIS-561nm 100mW, OBIS-639nm 70mW, and used the 2nd generation LaVision beam combiner. The images were acquired with an Andor CMOS sNEO camera. Main acquisitions were done with the LVMI-Fluor 4X/O.3 WD6 LaVision Biotec objective. With this objective, horizontal focusing mode was used, with a light sheet NA = 0.1 and 2μm step size.

For low-magnification acquisitions (autofluorescence and whole brain overviews), the objective is changed to a MI PLAN 1.1X/0.1 for the reference scan at 488nm excitation (tissue autofluorescence) or 639nm for axons, using horizontal focusing, 6μm step size, and light sheet numerical aperture to 0.03 NA.

#### Data analysis

##### Computing resources

The processing with ClearMap was done on local workstations, either Dell Precision T7920 or HP Z840. Each workstation was equipped with 2 Intel Xeon Gold 6128 3.4G 6C/12T CPUs, 512Gb of 2666MHz DDR4 RAM, 4x1Tb NVMe Class 40 Solid State Drives in a RAID0 array (plus a separate system disk), and an NVIDIA Quadro P6000, 24Gb VRAM video card. The workstations were operated by Linux Ubuntu 20.04LTS. ClearMap 2.0 was used on Anaconda Python 3.7 environment. Imaris 9 (Oxford Instruments) was used for qualitative analysis and data visualization.

##### TrailMap and TubeMap axon segmentation

Stitched raw images were segmented with TrailMap^18^,^52^ and a binary mask generated using a probability threshold of 0.5. The binary mask was then processed through TubeMap^51^ to align the axon pixels above the probability threshold to the Allen Brain Institute CCFv3 reference atlas. Quantifications of axon densities were obtained from the coordinates of both branching and non-branching points of the graph.

##### Single axon tracing

Single axon reconstructions were done manually using virtual reality-assisted tracing (SyGlass, IstoVision). The neuron reconstructions were exported in the .SWC format and imported in ClearMap ^16^ to align them to the reference brain atlas.

##### 3D analysis of collaterals in the brainstem

Neural projections in the brainstem were segmented using TrailMap.^18^ TrailMap output was binarized from the probability image with a threshold of 0.95. The coordinates of the positive pixels were downsampled to a 25μm isotropic resolution. Next, a mask of the hindbrain was generated from a downsampled autofluorescence image (25μm isotropic, imaged with 488 nm) using ITKsnap (http://ITKsnap.org)^50^, and applied to the downsampled coordinates from the binarized image. To compare axon densities across each hemisphere, a 3D plane (ax + by + cz + d = 0) was calculated based on a set of three points coplanar to the midline. To exclude the axons from the cortico-spinal tract, two planes of sagittal orientation, parallel to the midline, were calculated, again based on the three points.

Finally, the density of positive axon pixels from the brainstem projections was used to calculate a lateralization index (LI): (n_ipsi_- n_contra_)/(n_ipsi_ + n_contra_) . If LI = 0 the collaterals are perfectly symmetrical, LI = -1 in the case of a maximal contralateral bias and LI = 1 in the case of a maximal ipsilateral bias relative to the injection site.

### Quantification and statistical analysis

All data are presented in figures as mean ± SEM. Experiments were randomized, and no statistical methods were used to predetermine sample size. Investigators were blind to the experimental conditions. Comparisons between two conditions were analyzed by unpaired Mann-Whitney ranked U test. All statistical tests used were two-tailed. Statistical analysis was performed using R through Python 3.8 (rpy2, scipy). Figures 4F, 5I, and 6G: Mann-Whitney tests.
