# Supplementary material for: Organization and development of bilateral somatosensory feedback projections in mice
Source: iScience. 2025 May 21;28(6):112725. doi: 10.1016/j.isci.2025.112725 (PMC12177178; doi:10.1016/j.isci.2025.112725)
Supplement: Supplementary file 2 — Document S1. Figures S1–S3 [file mmc1.pdf]

iScience, Volume *28*

Supplemental information

Organization and development of bilateral somatosensory feedback projections in mice

Grace Houser, Alba Vieites Prado, Thomas Topilko, Clara Nguyen, Patricia Gaspar, and Nicolas Renier

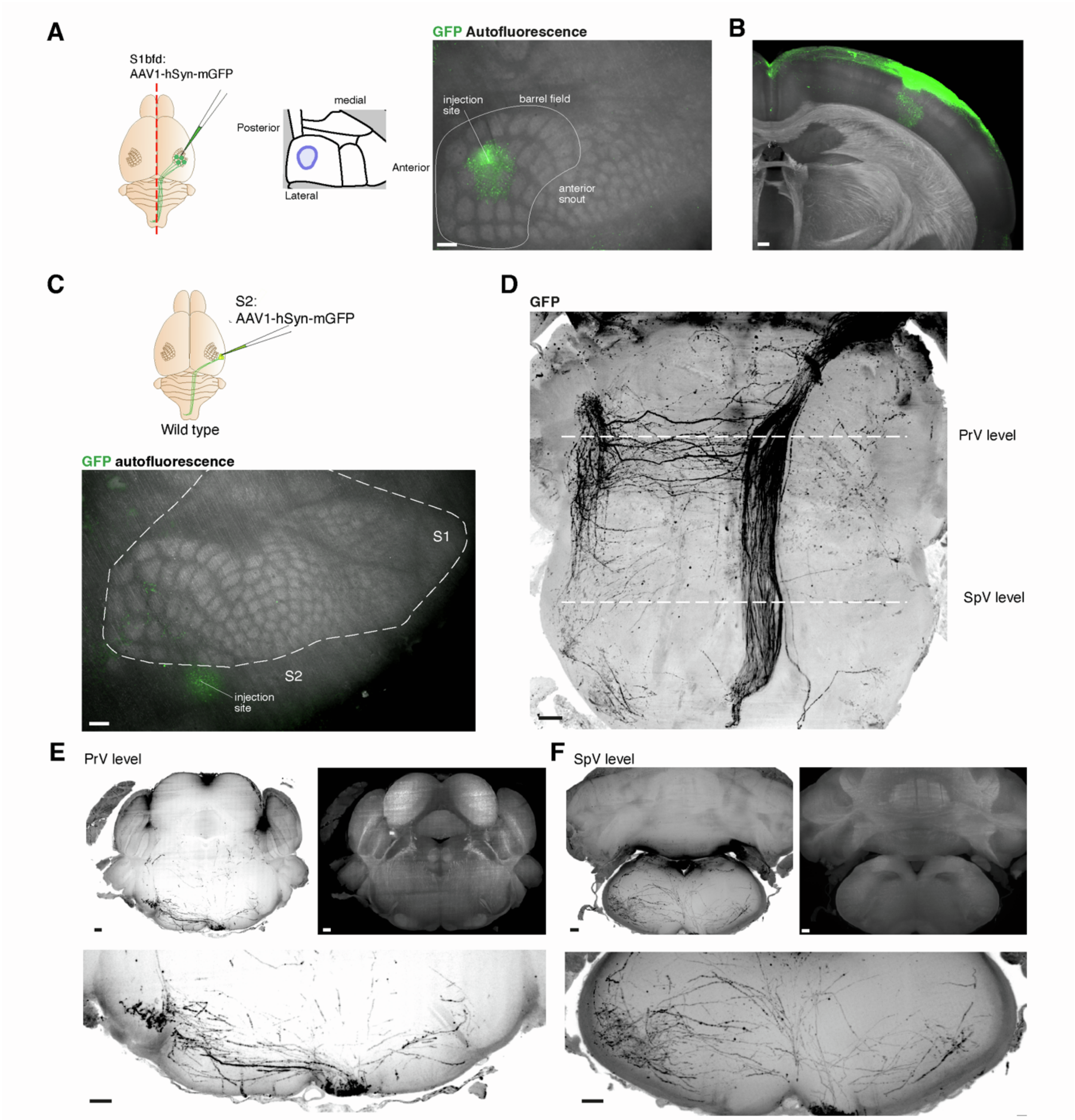

Supplementary Figure 1, related to Figure 1: Organization of cortico-bulbar projections from supplemental somatosensory areas

A-B 3D maximum intensity projection of the autofluorescence signal (grey) and GFP signal (green) in somatosensory areas in an adult iDISCO+ cleared brain in a top cortical projection (A) or coronal projection (B). Injections were made in the barrel field, at the level of rows C and E. The injection spread is outlined on the CCFv3 annotation map.

C 3D maximum intensity projection of the autofluorescence signal (grey) and GFP signal (green) in somatosensory areas in an adult iDISCO+ cleared brain. The brain was injected at the border of the supplemental somatosensory cortex (S2), lateral to the limit of the A-row barrels in the barrel cortex.

D Ventral brainstem projection of the GFP+ descending axons from S2-injected cortical neurons, showing a contralateral projection to the trigeminal complex, and very sparse ipsilateral collaterals. The dashed lines indicate the projection levels shown in C and D.

E-F 100µm coronal projection at the level of the PrV (C) or SpV (D) of the GFP labeled collaterals, showing many contralateral projections, and a few axons projecting to the ipsilateral trigeminal complex. Scale bars are 200µm.

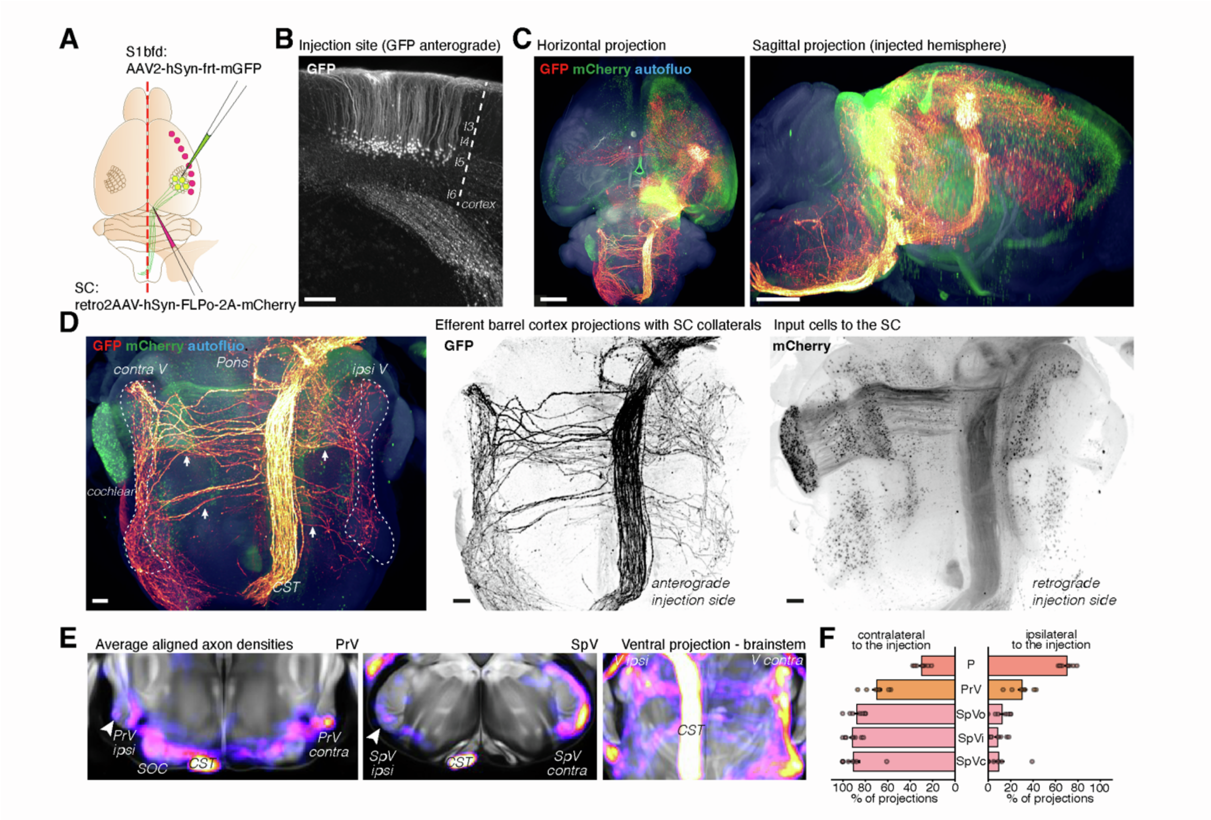

Supplementary Figure 2, related to Figure 5: Dual viral targeting of layer 5 neurons show neurons projecting to both the ipsilateral brainstem and ipsilateral Superior Colliculus (SC)
A Strategy for the selective targeting of layer 5 cortical neurons. GFP+ neurons are seen in the layer 5 of the barrel cortex (100µm transverse projection of a whole-brain scan) following a dual AAV injection targeting the barrel cortex neurons projecting to the SC.
B Detail of the anterograde injection site, showing layer 5 restricted labeling of pyramidal neurons.
C Whole brain projections of dual-AAV injected brains showing the patterns of input cells to the SC (green), SC-connected efferent projections of the barrel cortex (red) and autofluorescence (blue).
D Zoom at the level of the brainstem (ventral view) showing the CST tract, the pyramid decussation and 2 levels of collaterals projecting to the trigeminal complex, consistent with the bulk tracings.
E Registered averaged voxel densities of axons segmented with TrailMap shown at different coronal levels highlighting the major projection sites of the SC-connected long-range efferent neurons of the barrel cortex (n=7).
F Quantification of the laterality of axon densities in the brainstem target nuclei of the somatosensory CST.
Scale bars are 200µm, except panel (C): 1mm

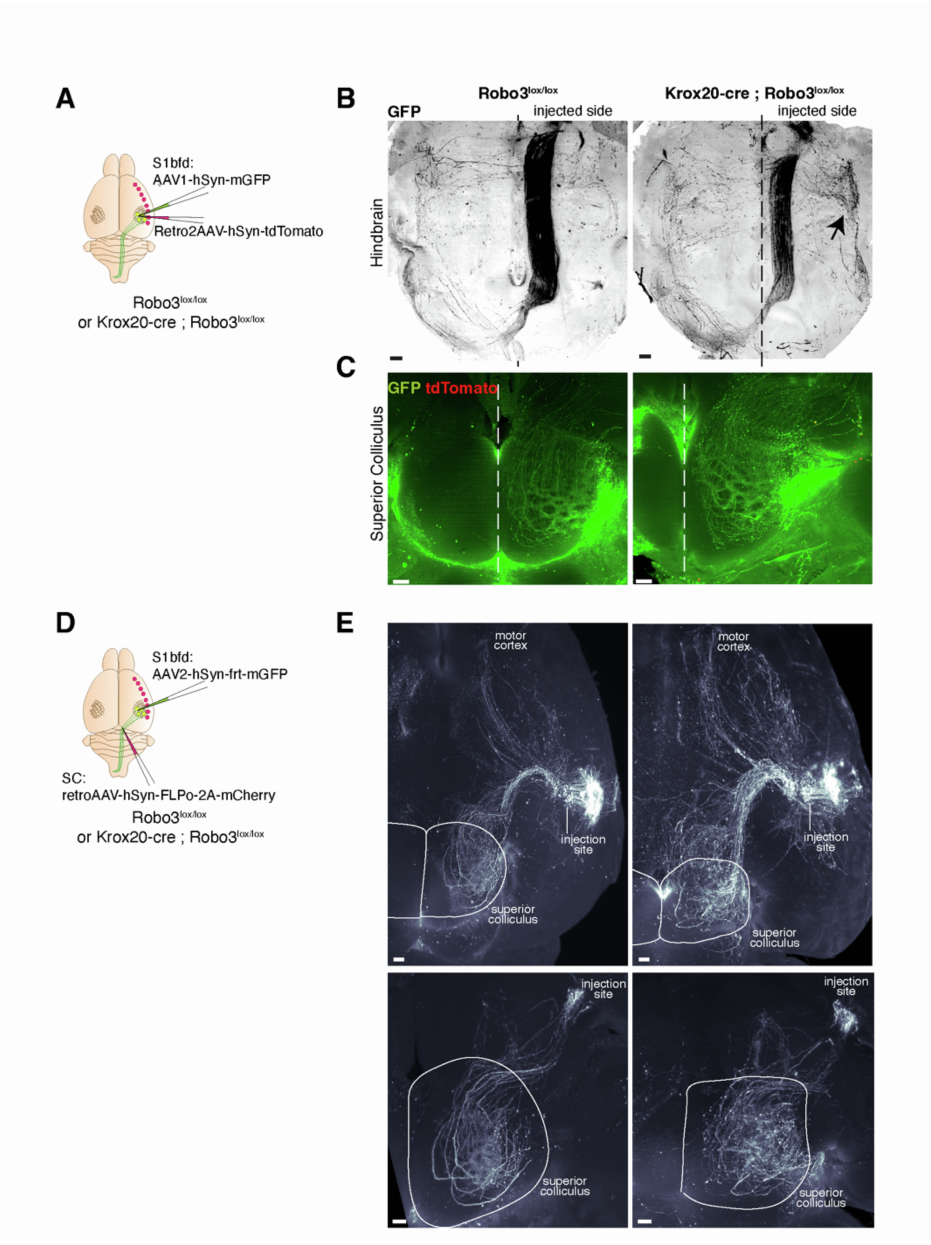

Supplementary figure 3, related to Figure 5: Control of the cortical projections to the superior colliculus in Krox20-cre ; Robo3$^{lox/lox}$ mutants

A Anterograde and retrograde viral injections in the barrel cortex of control Robo3$^{lox/lox}$ and mutant Krox20-cre ; Robo3$^{lox/lox}$ mice.

B Ventral projections of the brainstem in Robo3$^{lox/lox}$ and Krox20-cre ; Robo3$^{lox/lox}$ mutants, showing the stronger ipsilateral projection to the trigeminal complex in the mutants (arrow).

C Dorsal projections of collaterals to the superior colliculus, showing similar lateralization and reticulation in controls and mutant mice. (n=3/3 controls/mutants).

D Dual viral injections in the barrel cortex and superior colliculus of Robo3$^{lox/lox}$ and Krox20-cre ; Robo3$^{lox/lox}$ mutants, targeting layer 5 neurons of the barrel cortex projecting to the superior colliculus.

E Dorsal projection of GFP+ neurons traced from the barrel cortex to the superior colliculus. Top panels show the full extent of dorsal axons, including the motor cortex, while the bottom panels focus on axons projecting to the superior colliculus. No differences are seen in the laterality of the collateral branches in the superior colliculus between controls and mutants. A few straddler axons contacting the side contralateral to the injection are visible on both control and mutant animals. (n=7/5 controls/mutants).
Scale bars are 200µm.
